# Chronic kidney disease causes blood-brain barrier breakdown via urea-activated matrix metalloproteinase-2 and insolubility of tau protein

**DOI:** 10.18632/aging.205164

**Published:** 2023-10-25

**Authors:** Hisazumi Matsuki, Shintaro Mandai, Hiroki Shiwaku, Takaaki Koide, Naohiro Takahashi, Tomoki Yanagi, Shunsuke Inaba, Saaya Ida, Tamami Fujiki, Yutaro Mori, Fumiaki Ando, Takayasu Mori, Koichiro Susa, Soichiro Iimori, Eisei Sohara, Hidehiko Takahashi, Shinichi Uchida

**Affiliations:** 1 Department of Nephrology, Graduate School of Medical and Dental Sciences, Tokyo Medical and Dental University, Bunkyo City, Tokyo 113-8519, Japan; 2 Department of Psychiatry and Behavioral Sciences, Graduate School of Medical and Dental Sciences, Tokyo Medical and Dental University, Bunkyo City, Tokyo 113-8519, Japan; 3 Center for Brain Integration Research, Tokyo Medical and Dental University, Bunkyo City, Tokyo 113-8519, Japan

**Keywords:** chronic kidney disease, cognitive impairment, blood brain barrier, proteomics, urea

## Abstract

Chronic kidney disease (CKD) causes cognitive impairment and contributes to the overall global burden of dementia. However, mechanisms through which the kidneys and brain communicate are not fully understood. We established a CKD mouse model through adenine-induced tubulointerstitial fibrosis. Novel object recognition tests indicated that CKD decreased recognition memory. Sarkosyl-insoluble-proteomic analyses of the CKD mouse hippocampus revealed an accumulation of insoluble MAPT (microtubule-associated protein tau) and RNA-binding proteins such as small nuclear ribonucleoprotein U1 subunit 70 (SNRNP70). Additionally, there was an accumulation of Immunoglobulin G (IgG), indicating blood-brain barrier (BBB) breakdown. We identified that expressions of essential tight-junction protein claudin-5 and adherens-junction protein platelet endothelial cell adhesion molecule-1 (PECAM-1/CD31) were decreased in the brain endothelial cells of CKD mice. We determined urea as a major uremic solute that dose dependently decreased both claudin-5 and PECAM-1 expression in the mouse brain endothelial cell line bEnd.3 cells. Gelatin zymography indicated that the serum of CKD mice activated matrix metalloproteinase-2 (MMP2), while marimastat ameliorated the reduction of claudin-5 expression by urea in bEnd.3 cells. This study established a brain proteomic signature of CKD indicating BBB breakdown and insolubility of tau protein, which are pathologically linked to Alzheimer's disease. Urea-mediated activation of MMP2 was partly responsible for BBB breakdown in CKD.

## INTRODUCTION

Chronic kidney disease (CKD) is an increasingly recognized public health burden, affecting nearly 700 million adults globally, with higher rates in more developed countries [1]. Patients with CKD suffer from various neurological complications, including anxiety, depression, motor abnormalities (restless leg syndrome), sleep disturbances, and cognitive impairment [2]. A number of studies reported that kidney-related risk factors, such as albuminuria and estimated glomerular filtration rate (eGFR), are independently associated with both dementia and mild cognitive impairment (MCI) [3–5]. These disorders are characterized by deficits in executive function, memory, and attention [5], thereby linking CKD with cognitive decline in the aging population. A recent study estimated that 10% of dementia cases could be attributed to a mild kidney dysfunction, as defined by reduced estimated glomerular filtration rate (eGFR <60 mL/min/1.73 m^2^) by fraction analyses [6]. However, the pathophysiology of the kidney–brain axis affecting the central nervous system is not fully understood, thus elucidating these mechanisms may provide novel insights into the pathophysiology of dementia and degenerative brain diseases.

The kidneys and the brain are the most highly perfused organs in the human body. Similar hemodynamics are observed in the vascular bed of glomeruli as well as in the brain [7]. In neurodegenerative diseases, such as the Alzheimer’s disease (AD), recent evidence suggests that blood–brain barrier (BBB) dysfunction is associated with the accumulation of several vasculo- and neurotoxic molecules within the brain parenchyma [8]. In tauopathies, such as AD, accumulation of insoluble tau is a primary cause of pathogenesis of the disease [9, 10]. More recently, accumulating evidence has indicated that a variety of proteins or peptides, in addition to tau, are enriched in the detergent-insoluble fraction of AD brain specimens [11–13].

Importantly, the link between AD pathogenesis and undefined vascular mechanisms other than traditional vascular dementia mediated by arterial hypertension, stroke, and microvascular injury of the brain remains unclear [9]. A previous study found that a high-salt diet promoted tau phosphorylation and accumulation of insoluble tau [14]. CKD results in the accumulation of both salt and water in the body and might be a risk factor correlated with AD onset and cognitive impairment [15]. A recent study identified that smaller hippocampal volumes in hemodialysis patients were associated with impaired cognition [16]. In addition, a recent Mendelian randomization study revealed that CKD alters the brain cortical structure, including a reduction in cortical thickness, indicating neurodegeneration due to kidney damage [17]. However, this “neurodegenerative hypothesis” in CKD-related dementia has yet to be proven [18]. To address these points, this study determined the soluble and insoluble-proteomic signatures in the CKD mouse hippocampus and found an accumulation of detergent-insoluble proteins and BBB disruption, which are phenotypes shared in AD pathogenesis. We also identified urea-mediated matrix metalloproteinase-2 (MMP2) activation as the key mechanism of BBB disruption in CKD.

## RESULTS

### CKD causes loss of recognition memory in mice

**Figure 1:**
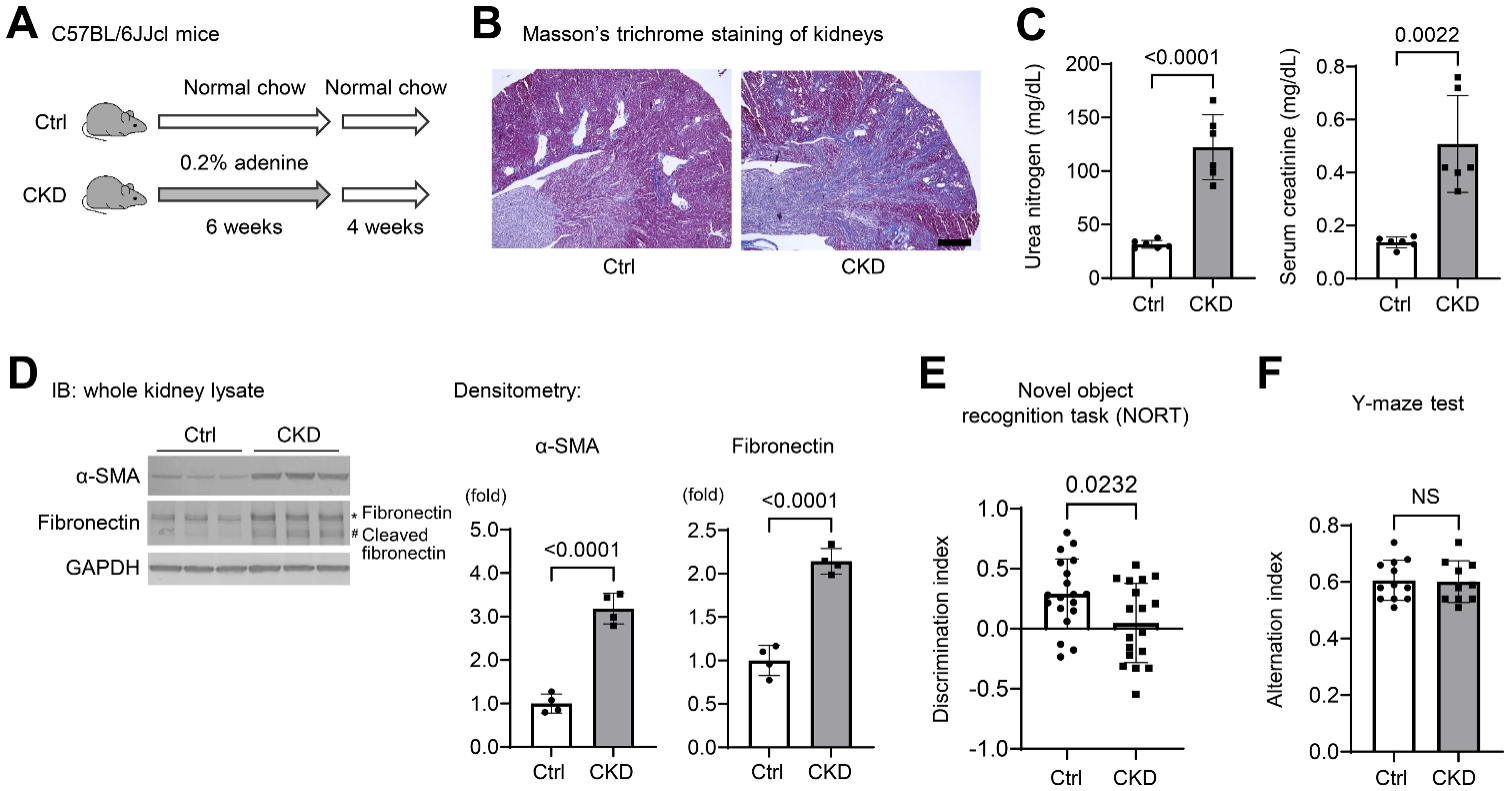
**Chronic kidney disease causes loss of spatial working memory in mice.** (**A**) Establishment of a CKD model in mice by providing wild-type C57BL/6JJcl mice a diet containing 0.20% adenine for six weeks. (**B**) Masson’s Trichrome staining showed tubulointerstitial fibrosis and diffuse tubular dilations in the kidneys of adenine-treated mice. Scale bar, 500 μm. (**C**) Serum urea nitrogen and creatinine, indicating uremic solutes, were elevated in CKD (*n* = 6 for each group). (**D**) The protein expression levels of α-smooth muscle actin (α-SMA) and fibronectin with cleaved fibronectin were elevated in the kidney tissues of CKD mice (*n* = 4 for each group). (**E**) We performed the novel object recognition test. The discrimination index, which represents a spatial reference memory, was significantly decreased in the CKD group compared with the control group (n = 19 in the control group; n = 18 in the CKD group). (**F**) The percentages of spontaneous alternation in the Y-maze test were not significantly different between the two groups (*n* = 12 in the control; n = 10 in the CKD group, respectively). Data are presented as mean ± standard deviation of the mean. Normality was assessed with the Shapiro–Wilk test. Statistical significance between the two groups was evaluated using an unpaired t test. *P* < 0.05 was considered statistically significant. CKD, chronic kidney disease.

To investigate the molecular mechanisms of cognitive decline in CKD, we established a CKD model in mice by adenine-induced tubulointerstitial fibrosis [19] (Figure 1A), and examined whether this model could influence the short-term memory of mice. Masson’s trichrome staining of CKD mouse kidneys indicated diffuse tubular dilatation and increased fibrotic areas (Figure 1B). Serum uremic solutes, urea nitrogen, and creatinine were also elevated in CKD mice (Figure 1C). The protein expression levels of α-smooth muscle actin (α-SMA), fibronectin, and cleaved fibronectin were elevated in kidney preparations from CKD mice (Figure 1D).

To examine the cognitive function of CKD mice, we performed neurobehavioral testing that included novel object recognition (NOR) and Y-maze tests [20, 21]. Using NOR, we found that the discrimination index, which represents short-term recognition memory, was significantly decreased in the CKD group as compared to the controls (*n* = 19 or 18; *P* = 0.0232) (Figure 1E). Pertaining to the spatial working memory, the percentages of spontaneous alternation in the Y-maze test were not significantly different between either group (Figure 1F). These findings suggest that cognitive function, spatial-reference memory, but not the spatial-working memory, was impaired after a relatively short-term exposure to adenine.

### Soluble and insoluble-proteomic analyses of CKD mouse hippocampus reveal the increase in a subset of proteins associated with BBB breakdown and AD

**Figure 2:**
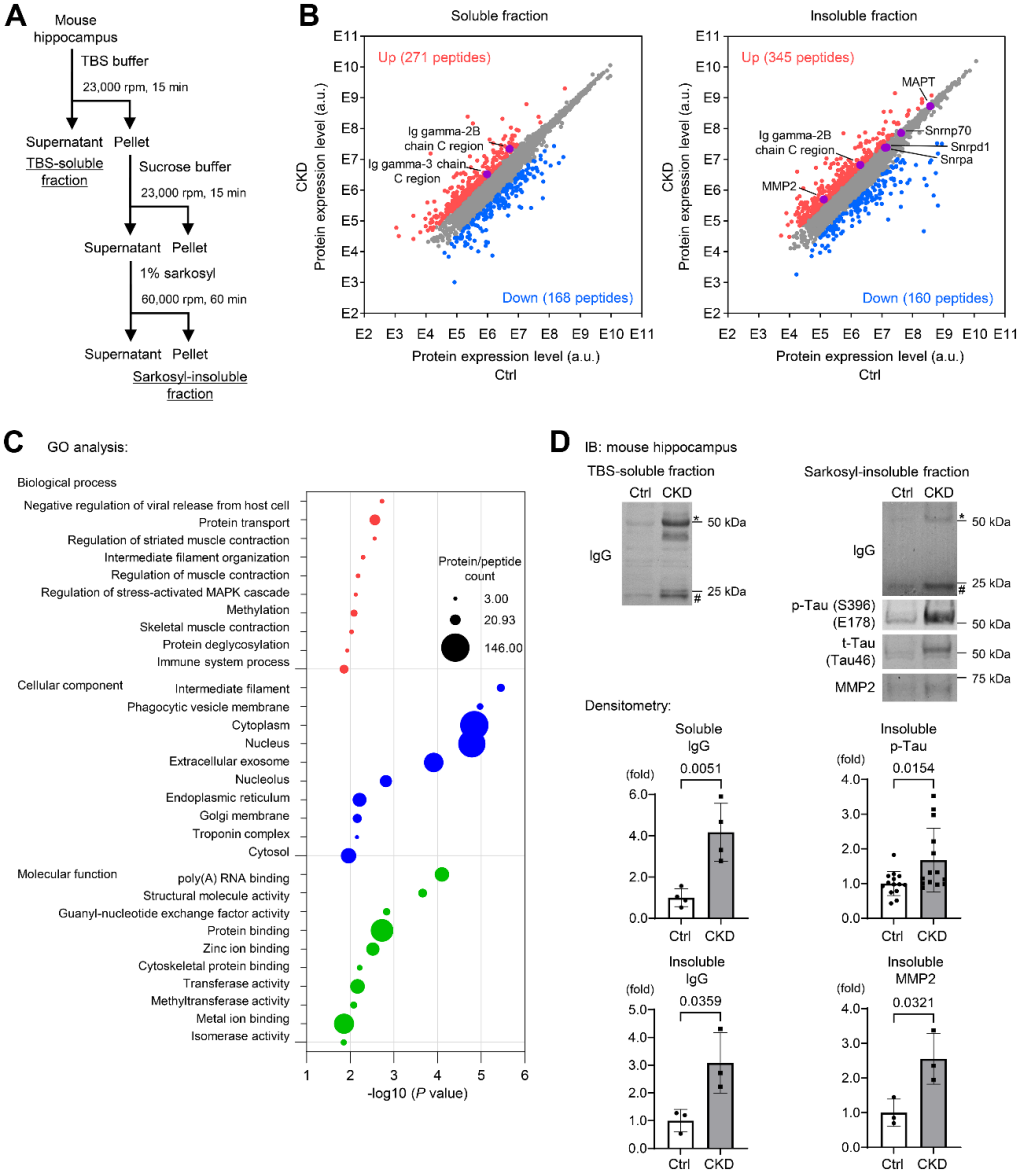
**Soluble and insoluble-proteomic analyses of mouse hippocampus reveal the increase in a subset of proteins associated with BBB breakdown and Alzheimer’s disease in CKD.** (**A**) Proteomic analyses using TBS-soluble and detergent-insoluble fractions of mouse hippocampus tissue lysates. We performed a mass spectrometry on the mixture of mice hippocampus tissue lysates in the CKD (n = 3) and control (n = 3) groups after a sequential biochemical extraction into TBS (salt buffer)-soluble fraction and sarkosyl (detergent buffer)-insoluble fraction. (**B**) Among the identified unique proteins or peptides over 7,000, we focused on upregulated (red) and downregulated (blue) DEGs in CKD when the fold changes were ≥3.0 compared to the controls. (**C**) The enrichment analysis of the DEGs in the insoluble fraction with the GO functional analysis revealed several pathways of interest, including “biological processes,” “cellular components,” and “molecular functions.” (**D**) Western blotting was performed to validate the proteomic analyses and showed that CKD increased expressions of soluble/insoluble IgG, and total and phosphorylated tau and MMP2 in the sarkosyl-insoluble (aggregated) fraction in the hippocampus. * Represents IgG heavy chain, and # represents the IgG light chain. The densitometry of IgG was determined with a mass of heavy and light chains. The densitometric analysis showed that CKD increased soluble IgG (n = 4 per group), insoluble IgG (n = 3 per group), insoluble phosphorylated tau (n = 14 per groups), and insoluble MMP2 (n = 3). Data are presented as mean ± standard deviation of the mean. Normality was assessed with the Shapiro–Wilk test. Statistical significance between the two groups was evaluated using an unpaired t test or Wilcoxon signed-rank test. When variables were nonparametric, we used the Wilcoxon signed-rank test. *P* < 0.05 was considered statistically significant. CKD, chronic kidney disease; MMP2, matrix metalloproteinase-2.

To determine a brain proteomic signature in CKD mice, we performed proteomic analyses of hippocampus tissue lysates from TBS-soluble and detergent-insoluble aggregated protein fractions [11–13] (Figure 2A), because neurodegenerative diseases currently encompass a broad range of disorders characterized by the deposition of insoluble protein aggregates, which contribute to disease progression [11]. We performed mass spectrometric analysis of mouse hippocampal tissue lysates prepared from TBS-soluble fraction as well as sarkosyl-insoluble fraction from three CKD and three control specimens [22]. We identified over 7,000 unique protein fragments or peptides that were differentially expressed ≥3.0 fold between CKD and control samples, and the differentially expressed genes (DEGs) are depicted in Figure 2B. Gene ontology (GO) enrichment analysis for all DEGs in the insoluble fraction revealed several pathways of interest, including “biological processes” (e.g., protein transport and intermediate filament organization), “cellular components” that were predominantly composed by organelles, and “molecular functions,” particularly poly(A) RNA-binding and protein binding (Figure 2C).

**Figure 3:**
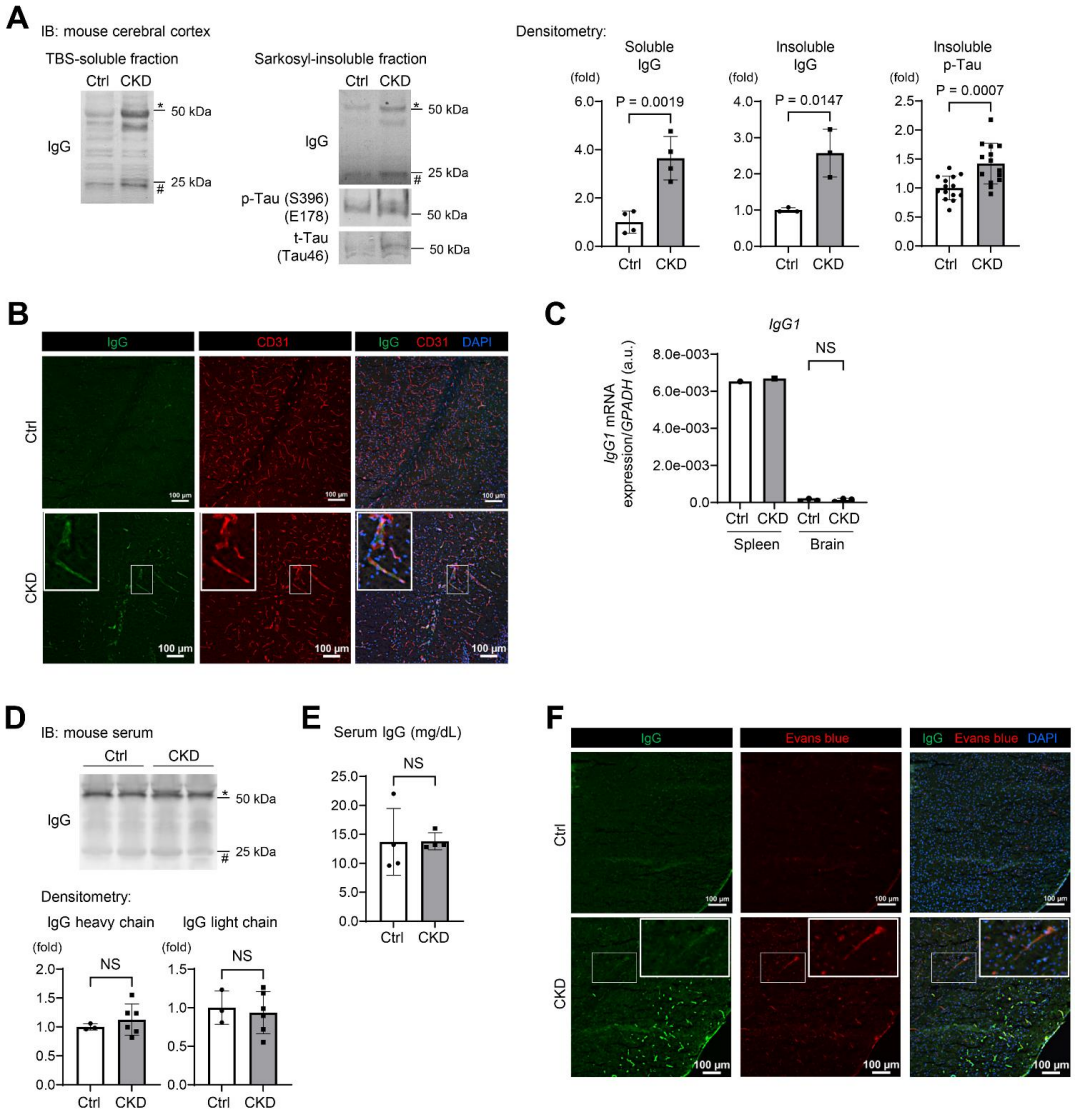
**CKD increases a leakage of IgG from cerebral blood vessels to the brain parenchyma in mouse hippocampus and cerebral cortex.** (**A**) Western blotting showed that CKD increased the expressions of soluble IgG (*n* = 4 per group), insoluble IgG (*n* = 3 per group), and phosphorylated tau (*n* = 14 per group) in the sarkosyl-insoluble (aggregated) fraction of the cerebral cortex. (**B**) The immunofluorescence study with a confocal microscopy showed scattered depositions of IgG in the subendothelial area of the hippocampus parenchyma. (**C**) Quantitative reverse transcription-PCR showed that the production of IgG1 mRNA in a brain tissue was negligibly low and not different between the CKD and control groups (*n* =3 per group). (**D**) Western blotting of serum from CKD and control mice showed no significant differences in IgG concentrations. (**E**) Measurements of serum IgG by turbidimetric immunoassay did not show differences between CKD and control mice. (**F**) Exogenously injected Evans-blue fluorescence was detected in CKD mouse cortex using a confocal microscopy, and was partly stained with IgG. Data are presented as mean ± standard deviation of the mean. Normality was assessed with the Shapiro–Wilk test. Statistical significance between the two groups was evaluated using an unpaired t test. *P* < 0.05 was considered statistically significant. CKD, chronic kidney disease; MMP2, matrix metalloproteinase-2.

This proteomic analysis also identified enrichment of detergent-insoluble tau (MAPT) (Figure 2B; right), a known aggregated protein in tauopathy [9, 10, 23, 24], with a wider exploration of proteins or peptides that were differentially expressed <3.0 fold between CKD and control groups. We also found that CKD increased the levels of insoluble RNA-binding proteins, such as small nuclear ribonucleoprotein U1 subunit 70 (SNRNP70) and other core U1 small nuclear ribonucleoproteins (snRNPs), which are strongly correlated to the pathology of tau insolubility in AD [11, 25, 26]. Immunoblotting experiments validated our proteomic analyses, as both total and phosphorylated tau were increased in the sarkosyl-insoluble (aggregated) fractions from the hippocampus (Figure 2D) and cerebral cortex (Figure 3A) of CKD mice. We further evaluated these findings in another model of CKD established by 5/6 nephrectomy [27] (Supplementary Figure 1A), which led to global tubulointerstitial fibrosis and an increase in uremic solutes (Supplementary Figure 1B, 1C). Tau insolubility was also observed in CKD mice after 5/6 nephrectomy (Supplementary Figure 1E).

The constituents of immunoglobulin G (IgG) heavy chains were markedly increased in both soluble and insoluble fractions (Figure 2B). Western blotting using mouse IgG alone as a secondary antibody clearly demonstrated the increased IgG expression in the CKD group in tissue lysates from the hippocampus in both soluble and insoluble fractions (Figure 2D). In addition to the hippocampus, the cerebral cortex within the CKD model also showed increased IgG expression (Figure 3A). The marked increase of IgG in brain tissues was replicated in the 5/6 nephrectomy CKD model (Supplementary Figure 1D). Assessment of leaky IgG with Western blotting of the brain lysates is a valid and efficacious way to evaluate BBB integrity [28–30]. Thus, these findings suggest that increased BBB flux is a key signature in the pathogenesis of the kidney–brain axis in CKD.

### CKD enhanced leakage of IgG from cerebral blood vessels to the brain parenchyma

To visualize whether CKD promoted IgG leakage, we performed immunofluorescence studies on mouse brain sections taken from CKD mice [31, 32]. Our adenine-induced nephropathy model showed scattered depositions of IgG in the subendothelial area of the hippocampus parenchyma (Figure 3B).

We therefore examined whether IgG elevation in CKD brain tissue originated from white blood cells (WBCs) localized in the brain or serum circulating IgG. Quantitative reverse transcription-PCR indicated that production of IgG1 mRNA in brain tissue was negligibly low and not statistically different between the CKD and control groups (Figure 3C). Western blotting of serum from CKD and control groups showed no significant differences in IgG concentrations (Figure 3D). In addition, measurements of serum IgG by turbidimetric immunoassay did not show differences between the groups (Figure 3E).

Further immunofluorescence studies revealed that exogenously injected Evans-blue dye [33] was only detected in CKD mice, and was partly stained with IgG (Figure 3F). These findings suggest that CKD is associated with extravascular leakage of proteins in circulation through disruption of the BBB.

We performed immunofluorescence staining for phosphorylated tau at S396 and β amyloid of the hippocampus of adenine-induced CKD model and control mice. As shown in Supplementary Figure 2, no obvious depositions were observed in both groups.

### The expression levels of tight-junction protein claudin-5 and adherens-junction protein PECAM-1/CD31 were decreased in the brain endothelial cells of CKD model mice

**Figure 4:**
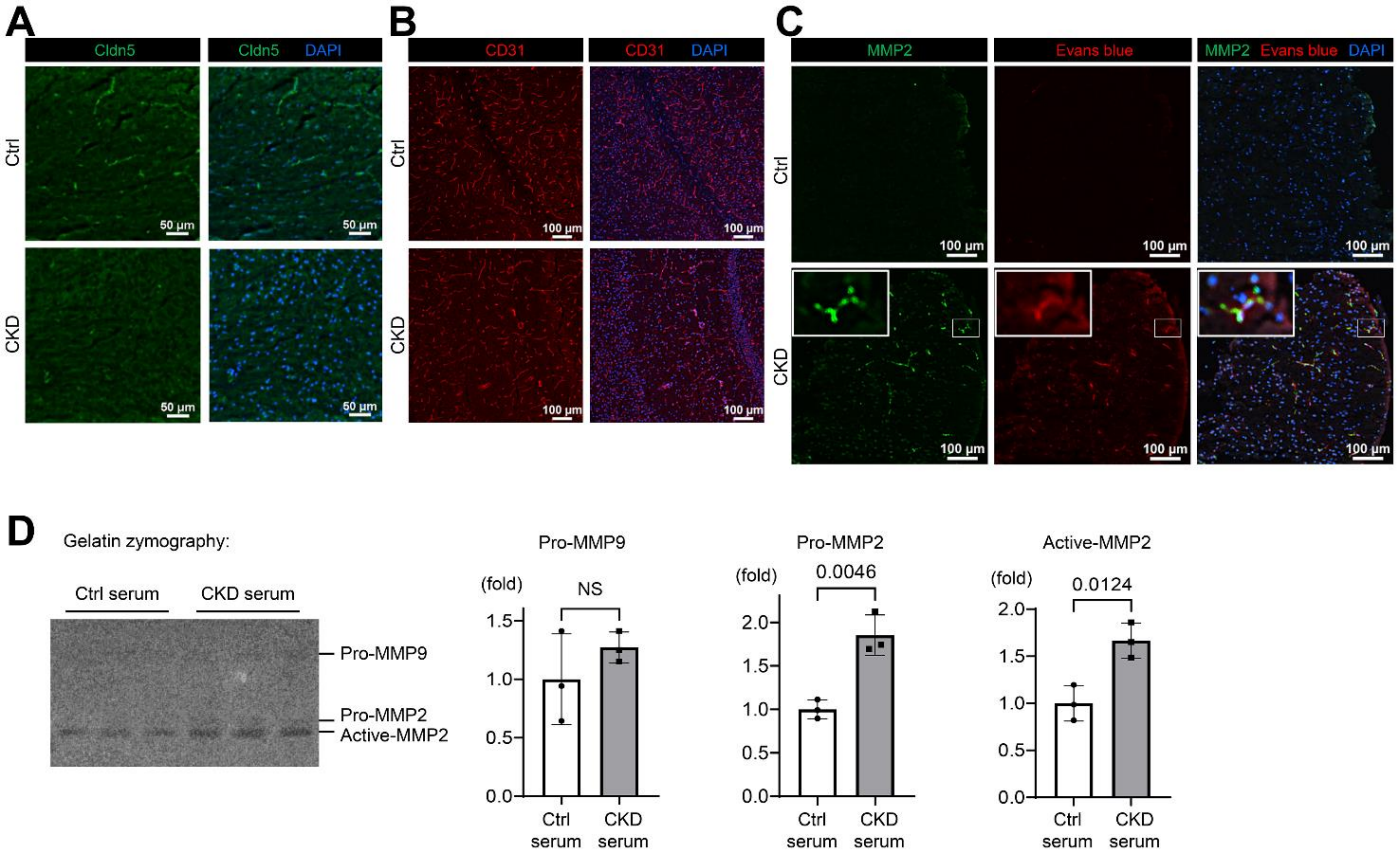
**CKD downregulates tight junction, adherens-junction, and basement membrane proteins in mice.** (**A**) Immunofluorescence indicating the staining pattern of claudin-5 in the hippocampus tissue of CKD mice compared to the control group. (**B**) Immunofluorescence study showing that the adherens-junction protein platelet/endothelial cell adhesion molecule-1 (PECAM-1)/CD31 expression was decreased in the hippocampus of CKD mice compared to the control group. (**C**) Immunofluorescence study showing that the protein expression of MMP2 was increased in the neocortex tissue of CKD mice, and partly stained with the extravasation of Evans blue dye. (**D**) We performed gelatin zymography using bEnd.3 cells treated with serum derived from CKD model mice. Uremic serum activated MMP2, but not MMP9 (*n* = 3 per group). Data are presented as mean ± standard deviation of the mean. Normality was assessed with the Shapiro–Wilk test. Statistical significance between the two groups was evaluated using an unpaired t test. *P* < 0.05 was considered statistically significant. CKD, chronic kidney disease; MMP2, matrix metalloproteinase-2.

To determine whether a loss of tight-junction proteins (TJPs) is attributable to CKD-induced BBB breakdown, we evaluated the expressions of claudin-5, the essential molecule regulating BBB integrity expressed in brain endothelium [34], with immunofluorescent staining using CKD model mice (Figure 4 and Supplementary Figure 3). As shown in Figure 4A and Supplementary Figure 3A, we identified that claudin-5 expression was sparsely present in brain tissues of CKD mice as compared to controls. We also found that adherens-junction protein platelet/endothelial cell adhesion molecule-1 (PECAM-1)/CD31 expression was decreased in the hippocampus of CKD mice (Supplementary Figures 3B, 3C, 4B). PECAM-1 contributes to the stability of adherens-junctions and the endothelial barrier via modulating the localization of vascular endothelial cadherin (VE-cadherin) and β-catenin [35].

We further investigated the role of MMP2, which was found to be increased in the detergent-insoluble fraction of CKD brain (Figure 2B), in the loss of claudin-5 and CD31, given that MMP2 is the major pathophysiological regulator of BBB integrity by modulating claudin-5 expression and distribution in endothelial cells [36, 37]. As shown in Figure 4C and Supplementary Figure 3D, protein expression of MMP2, which is a type IV collagenase [38], was increased in CKD in contrast to the decreased CD31 expression. MMP 2 was partly stained with the extravasation of Evans blue dye, suggesting the pathological link with activated MMP2 and a leaky BBB. We also evaluated the expression of collagen IV, a key component of the basement membrane, and found that it was suppressed in CKD hippocampus tissues (Supplementary Figure 3E).

To elucidate whether uremic serum in circulation could upregulate MMP2 activity in brain endothelial cells, we performed gelatin zymography, using the murine brain endothelial cell line bEnd.3 treated with serum derived from CKD model mice. As shown in Figure 4D, uremic serum activated MMP2 as opposed to MMP9 that did not. These findings suggest that CKD degrades TJPs and adherens-junction proteins, which might be regulated by MMP2 activation, leading to BBB breakdown.

### Urea downregulates claudin-5 partly via activation of MMP2 in cultured brain endothelial cells

To determine uremic solutes or toxins propagating from circulation that affect the protein expression levels of claudin-5 and CD31, we examined the effects of indoxyl sulfate (IS), trimethylamine N-oxide (TMAO), and urea on bEnd.3 cells.

**Figure 5:**
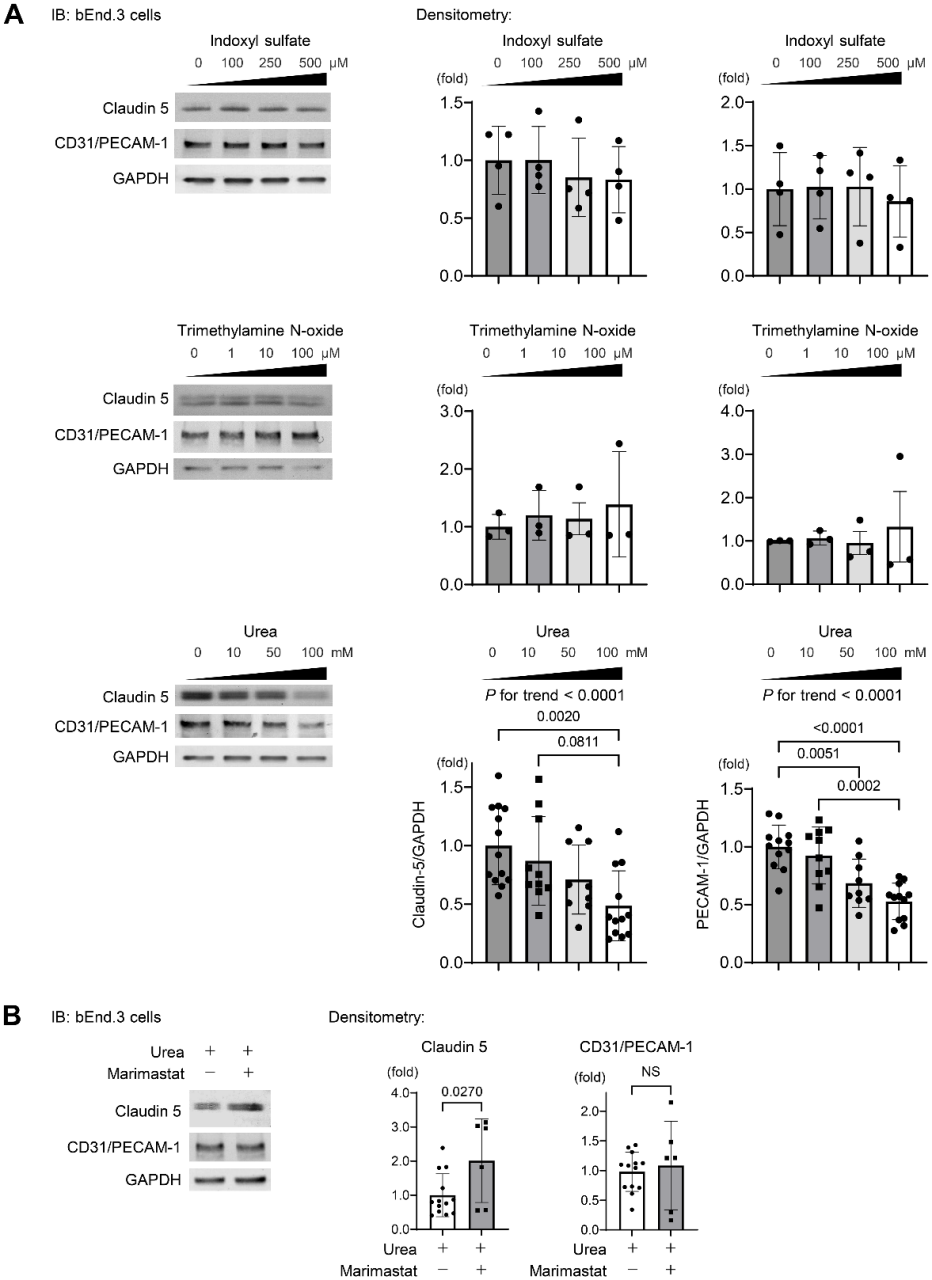
**Urea activates MMP2 leading to degradation of TJPs.** (**A**) Western blotting indicating the abundance of claudin-5 and CD31 after indoxyl sulfate (IS), trimethylamine N-oxide (TMAO), and urea treatment of bEnd.3 cells. IS and TMAO did not influence the expressions of claudin-5 and CD31, while urea suppressed both proteins dose dependently after treating bEnd.3 cells for 24 h (*n* = 13, 10, 9, or 12 per group, respectively). Statistical analyses among multiple groups were performed using one-way ANOVA, followed by Turkey’s post-hoc test for parametric variables or the Kruskal–Wallis test followed by Dunn’s multiple comparisons test for nonparametric variables. The Jonckheere–Terpstra trend test was used to indicate potential protein expression trends in response to a concentration gradient of uremic solutes. (**B**) We treated the cells with urea and a MMP inhibitor marimastat at 5 μM as the final concentration, which is the first MMP inhibitor used for clinical trials to suppress tumor growth in various cancers. Western blotting showed that the suppression of claudin-5 expression was ameliorated with marimastat, whereas CD31 expression remained unchanged (*n* = 13 in non-marimastat group; *n* = 6 in marimastat-treated group). Data are presented as mean ± standard deviation of the mean. Normality was assessed with the Shapiro–Wilk test. *P* < 0.05 was considered statistically significant. CKD, chronic kidney disease; MMP2, matrix metalloproteinase-2.

As shown in Figure 5A, IS and TMAO were not found to influence the expressions of claudin-5 and CD31, while urea suppressed dose dependently both proteins after treating bEnd.3 cells for 24 h. MMP-2 has been reported to regulate TJPs, basement membrane proteins, and PECAM-1 as substrates [36] and is involved in BBB breakdown via disruption of TJPs in several neurological diseases such as epilepsy and ischemic stroke [31, 34, 37, 39]. We treated bEnd.3 cells with urea and the MMP inhibitor marimastat, which is used in clinical trials to suppress tumor growth of various cancers [40]. The suppression of claudin-5 expression was partly recovered by marimastat, whereas CD31 expression remained unchanged (Figure 5B). These findings indicate that urea regulates the claudin-5 expression partly via MMP2 activation and is also involved in maintaining the integrity of BBB.

### A BBB disruption in the mouse hippocampus following elevated serum urea preceded the accumulation of insoluble tau in CKD

To clarify the time sequence of the events in CKD mouse brain including increased insoluble tau, BBB disruption, and increased serum urea, male C57BL/6 mice were exposed to different allocation periods of a 0.20%-adenine diet (2 days, 8 days, and 15 days) (Supplementary Figure 4A).

We removed the hippocampus and evaluated for soluble IgG, indicative of BBB disruption, and insoluble phosphorylated tau with Western blotting in each group. Blood tests showed that serum urea level started to increase 8 days after the exposure to 0.20%-adenine diet (Supplementary Figure 4B). Western blotting showed the increased soluble IgG heavy and light chains of mouse hippocampus 15 days after initiating a 0.20%-adenine diet (Supplementary Figure 4C). Within this time course, insoluble phosphorylated tau was not accumulated in the CKD mouse hippocampus (Supplementary Figure 4D). These findings suggest that the disruption of BBB followed the elevated serum urea in the early stage of kidney dysfunction, and the BBB disruption precedes the increase in insoluble tau under the chronic phase.

### Serum urea nitrogen is associated with a greater risk of cognitive impairment in patients with CKD

To elucidate factors linked to risk of cognitive impairment, we performed logistic regression analyses to assess the association of demographic and biological data, including serum urea nitrogen, with cognitive impairment using the two CKD cohorts [41]. We totally enrolled 980 CKD adults. Dialysis-independent CKD was defined according to the Kidney Disease Improving Global Outcomes (KDIGO) classification [42]. GFR was estimated using the three-variable Japanese GFR equations developed by the Japanese Society of Nephrology [43].

**Figure 6:**
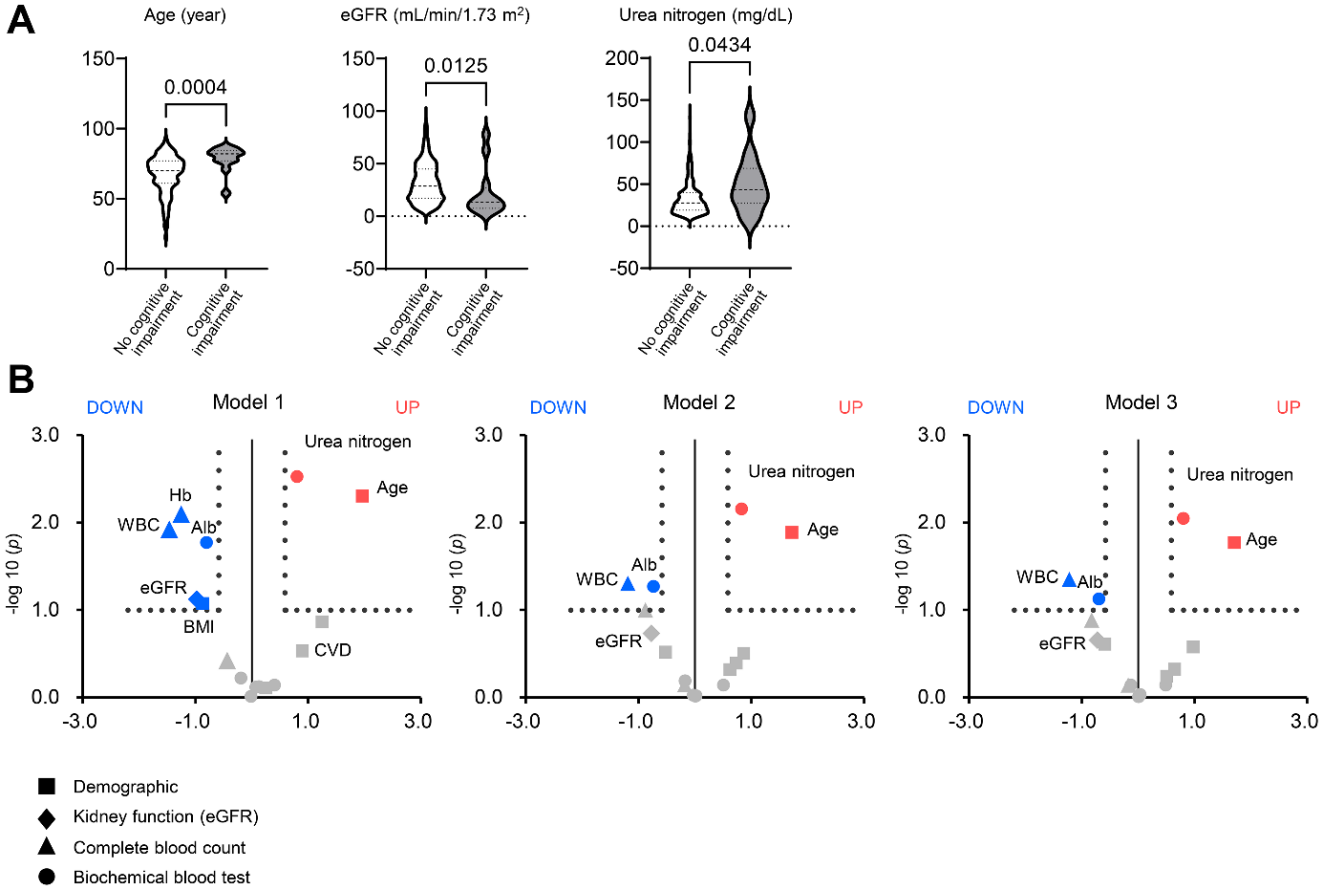
**Factors associated with cognitive impairment in patients with chronic kidney disease.** (**A**) Violin plots of age, eGFR, and urea nitrogen showing differences between patients with and without cognitive impairment in the two CKD cohorts consisting of 980 adults. The bold or thin dashed lines indicate medians or inter-quartile ranges. Normality was assessed with the Shapiro–Wilk test. Statistical significance between the two groups was evaluated using the Wilcoxon signed-rank test. (**B**) The volcano plot graphs show the associations of the demographic (square), kidney function (eGFR; diamond), complete blood count (triangle), and biochemical data (circle) including serum urea nitrogen with a risk of cognitive impairment in patients with chronic kidney disease (CKD). Using the two CKD cohorts, we performed univariate and multivariable logistic regression analyses. The horizontal line indicates that the threshold of odds ratios (ORs) was 1.5, and the vertical line indicated that the threshold of *P* value of logistic regression models was 0.1. Red spots represent factors increasing the risk of cognitive impairment, and blue spots represent factors mitigating cognitive impairment. Model 1: univariate (left). Model 2: adjusted for age and sex (middle). Model 3: Model 2 plus BMI, diabetes mellitus, and cardiovascular disease. BMI, body mass index; CKD, chronic kidney disease; OR, odds ratio.

As shown in Figure 6A, patients with cognitive impairment had lower eGFR expression and higher urea nitrogen levels than those without cognitive impairment. Figure 6B illustrates the volcano plot graphs showing the associations of the demographic (square), kidney function (eGFR; diamond), complete blood count (triangle), and biochemical data (circle) with risk of cognitive impairment. In the univariate analysis (left; model 1), 1-standard deviation (S.D.) increases in eGFR (18.7 mL/min/1.73 m^2^), WBC (1,894/μL), hemoglobin (2.26 g/dL), serum albumin (0.64 g/dL), and BMI (4.13 kg per m^2^) were associated with a lower risk of cognitive impairment. In contrast, 1 S.D increases in urea nitrogen (19.6 mg/dL) and age (13.7 years) were associated with a greater risk of cognitive impairment. After adjusting for age and gender (middle; model 2), and additionally BMI, diabetes mellitus, and cardiovascular disease (right; model 3), the association of eGFR with cognitive impairment did not remain significant [odds ratio (OR), 0.61; 95% confidence intervals (95% CI), 0.27–1.35 in model 3].

Elevated serum urea nitrogen was associated with a risk of cognitive impairment even after adjusting for confounders (OR, 1.74; 95% CI, 1.15–2.65), suggesting the greater impact of accumulated urea or uremic solutes on cognitive function rather than kidney function. Higher WBC (OR, 0.43; 95% CI, 0.19–0.98) and serum albumin (OR, 0.43; 95% CI, 0.36–1.05) were linked to a lower risk of cognitive impairment after adjustment. The effect of serum albumin on cognitive impairment indicates nutritional status as a mitigating factor.

**Figure 7:**
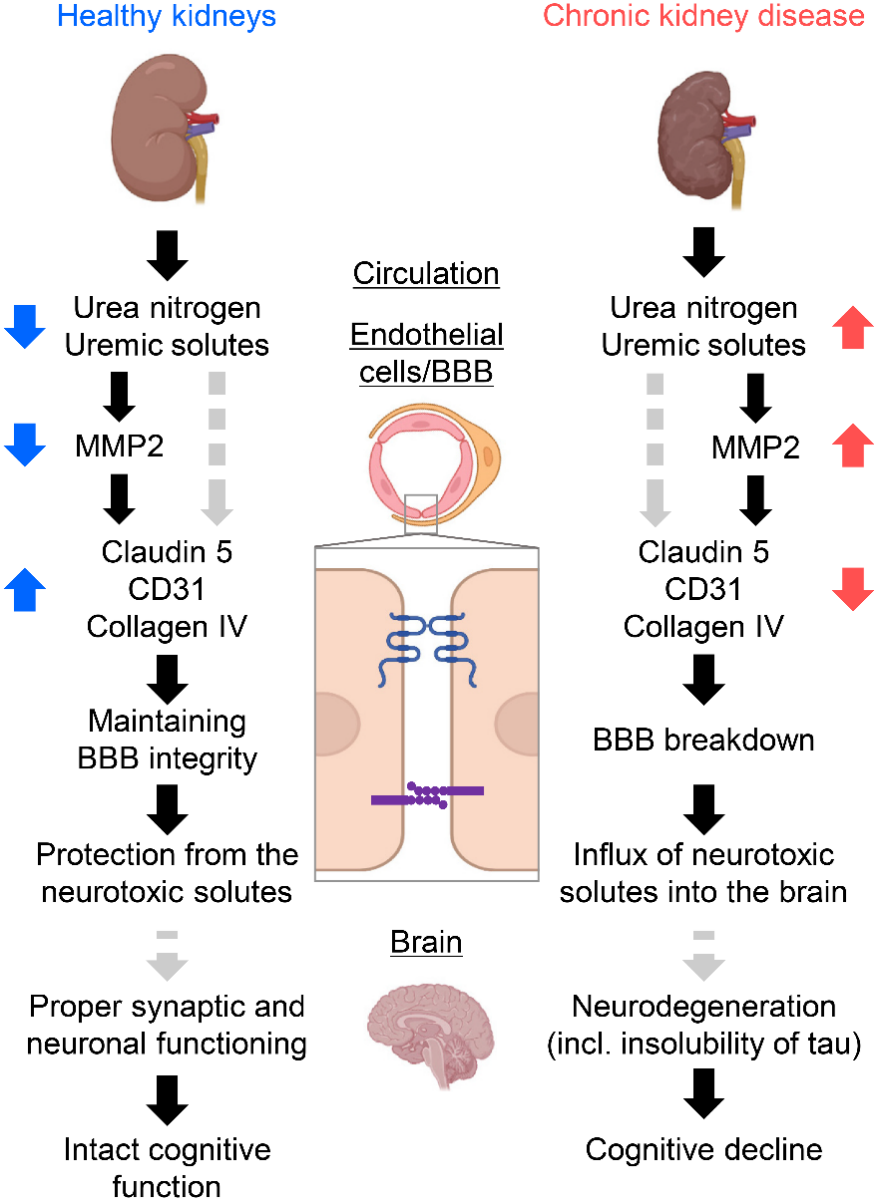
**Schematic summary of the study forming the kidney–brain axis.** Healthy kidneys sufficiently filtrate and excrete uremic solutes and toxins into urine. Thus, expressions of tight-junction proteins (TJPs) and adherens-junction proteins were sustained, offering fine tuning of the blood–brain barrier (BBB) and proper synaptic and neuronal functioning. Subsequently, the influx of neurotoxic solutes due to BBB opening causes neurodegeneration. The accumulation of insoluble tau or RNA-binding proteins similar to Alzheimer’s disease might result in cognitive decline in CKD. CKD, chronic kidney disease; MMP2, matrix metalloproteinase-2. Created with BioRender.com.

Our findings are summarized in Figure 7 and indicate a previously uncovered brain–kidney axis originating from BBB breakdown. CKD causes the accumulation of uremic solutes, particularly urea, which do not normally accumulate in healthy individuals. Urea induces active MMP2 in brain endothelial cells to degrade the claudin-5, PECAM-1, and collagen IV substrates, resulting in the influx of neurotoxic solutes due to BBB opening and worsening neurodegeneration. The accumulation of insoluble tau or RNA-binding proteins similar to the AD might result in cognitive impairment in CKD.

## DISCUSSION

The present study revealed the brain proteomic signature of CKD and confirmed the accumulation of insoluble tau and BBB disruption in the cerebral cortex and hippocampus, using CKD model mice displaying cognitive impairment. These findings were similar to those observed in neurodegenerative diseases, including the AD. We also found that the protein expressions of claudin-5, PECAM-1/CD31, and collagen IV were suppressed in the hippocampus of CKD mice. These results suggest that the structures of the BBB were compromised leading to an increase in the flux of solutes across the BBB. Compromised barrier property was partly regulated by urea-activated MMP2. We further showed that serum urea was more strongly associated with cognitive impairment compared to eGFR and a history of cardiovascular disease. These findings might bring novel insights into the pathophysiology of the brain–kidney axis underlying cognitive impairment in CKD and regulation of the flux across the BBB.

This study revealed the previously uninvestigated proteomic profiling of CKD brain using biochemical differential extraction. Evolving proteomic technologies have explored clinical AD specimens to elucidate the underlying molecular mechanisms of the disease. Subproteome analysis, such as amyloid plaque proteome, has evolved as an effective strategy compared to whole proteome analysis. Additionally, biochemical differential extraction of AD specimens offers an alternative means of enriching the aggregate proteome due to its low solubility [13]. This study is the first to perform insoluble-proteomic analysis of CKD hippocampus and revealed an increase in a subset of molecules shared with AD, including tau and RNA splicing proteins. In AD, tau proteins are hyperphosphorylated and abnormally folded, forming neurofibrillary tangles which are required for its pathological diagnosis [10, 24]. snRNP70 and additional spliceosome components also accumulate and form detergent-insoluble aggregates that are strongly correlated with amyloid-β and tau insolubility in AD and MCI [11, 25, 26]. RNA splicing dysfunction in concomitant insoluble snRNPs aggregates and in tauopathy was also reported [25, 44]. Our findings support the “neurodegenerative hypothesis” in CKD-related dementia [18]. Immunofluorescence staining for phosphorylated tau at S396 and β amyloid revealed no obvious depositions. Insoluble tau and β amyloid can be detected by immunofluorescence staining of rodent brains usually in human tau or β amyloid transgenic models. Further studies of the brain of CKD patients are necessary.

Since Zlokovic [8] proposed a pathological theory suggesting that cerebrovascular dysfunction and disruption in the neurovascular integrity contributes to the onset and progression of cognitive decline, microvascular contributions to cognitive impairment are increasingly recognized in AD [45, 46]. In the present study, we found an increase in IgG heavy chain in the CKD hippocampus, which may indicate BBB disruption. A recent study reported that BBB breakdown is an early biomarker of human cognitive decline, independent of amyloid-β and tau [47]. A BBB breakdown in CKD may be a background for the development of the common pathology seen in AD. Additionally, uremic toxins are reported to pass through the BBB; however, BBB disruption in CKD would be expected to promote this tendency, namely the influx of circulating pathogenic molecules. Consequently, this suggests a CKD-specific pathophysiology that may promote neurodegeneration. This speculation on pathogenesis in CKD mouse brain is supported by the time sequence of the events characterized by increased BBB flux and the subsequent accumulation of tau following elevated serum urea (Supplementary Figure 4).

The analysis of the brain whole lysate does not lead to a definitive conclusion, given that the brain vasculature accounts only for 0.1% (v/v) of the brain. In contrast, decreased expression of BBB component proteins was observed histologically. The previous reports showed the decreased protein expressions of TJPs (claudin-1, occludin, zonula occludens-1) in the colonic mucosa in CKD model rat and the decreased expression of TJPs (claudin-5, occludin, Junctional adhesion molecule-1) in immunofluorescent staining of subcutaneous fat biopsies from CKD patients [48, 49]. However, the mechanisms remain unknown. To investigate the molecular mechanisms and functional molecules in circulation that are essential for CKD-induced BBB disruption, we cultured bEnd.3 cells with urea and uremic toxins IS and TMAO, and found that the expressions of claudin-5 and PECAM-1 were decreased with urea in a concentration-dependent manner. Although previous studies have reported that urea-induced a disruption of the tight junction and barrier function of mouse cerebral vascular endothelial cells and human enterocytes, the molecular mechanisms have remained unknown [50, 51]. In this study, we focused on MMP-2, which has been reported to play an important role in the pathogenesis of BBB disruption in many neurological diseases, such as epilepsy, ischemic stroke, and dementia [37–39]. A recent study that used the 3D *in vitro* models of AD reported a correlation between the upregulation of MMP-2 and the downregulation of claudin-5 [52].

MMP-2 expression was indeed increased in the hippocampus of adenine-induced CKD mice histologically, and cell culture studies showed that exposure to CKD mice serum increased MMP-2 activity in bEnd.3 cells. Additionally, the MMP inhibitor marimastat [40], ameliorated the urea-induced decreased expression of claudin-5 in bEnd.3 cells. Increased serum levels of MMP-2 have also been reported in CKD patients, and a pathogenic role in renal interstitial fibrosis, possibly through the induction of epithelial mesenchymal transition, has been suggested [53]. These findings reveal the therapeutic potential targeting of MMP2 in treating CKD and CKD-related cognitive impairment, although further investigations are needed to elucidate the remaining factors resulting in decreased claudin-5 and CD31 expression.

The previous study showed that urea osmotically opens BBB, although transient BBB opening and influx of serum into the central nervous system (CNS) did not seem to cause neurological deficits [54]. Using CKD cohorts, we found the stronger correlation between urea nitrogen and cognitive impairment in CKD patients, in contrast to the weaker association between eGFR and cognitive impairment. This finding suggests that urea and additional solutes directly regulate CNS function rather than secondary factors due to low eGFR levels. Several studies showed a sharp collinearity between higher urea nitrogen level and lower white matter integrity in patients with end-stage kidney disease (ESKD) [55]. Accumulating experimental and clinical data demonstrated the direct toxicity of urea [56]. Urea might be one of direct contributors that affect cognitive impairment via a BBB breakdown and greater chronic exposure of CNS to uremic serum. The avoidance of dehydration and high dietary protein intake, which lead to elevated serum urea, may protect cognitive function in CKD. As to whether correction of uremia can mitigate cognitive impairment in CKD patients or not, further randomized clinical trials are needed.

In conclusion, this study demonstrated the brain TBS-soluble and detergent-insoluble-proteomic signatures characterized by insolubility of tau and BBB breakdown similar to neurodegenerative diseases. We further found that uremic solutes, particularly urea, downregulates protein expressions of TJPs and adherens-junction proteins that are essential for maintaining BBB integrity, partly via activation of MMP2.

## MATERIALS AND METHODS

### Cell lines and cell culture

The ATCC® CRL-2299™-immortalized mouse brain-derived endothelial cell line (bEnd.3; ATCC; Manassas, VA, USA) was cultured in Dulbecco’s Modified Eagle’s Medium (DMEM) (ATCC) supplemented with 10% fetal bovine serum. The cells were cultivated at 37° C under 5% CO_2_ condition in a humidified incubator. The cells were used at passages 5–15, at which time they were 80% confluent.

### Administration of marimastat and uremic solutes, or toxins in the cells

bEnd.3 cells were grown in 6-well plates at 50% confluency. The cells were treated with uremic toxins [urea (Sigma-Aldrich, St. Louis, MO, USA), indoxyl sulfate (Sigma-Aldrich), and Trimethylamine N-Oxide Dihydrate (TCI, Japan)] dissolved in phosphate buffered saline (PBS). For marimastat treatment, marimastat (BB-2516, Selleck) dissolved in 100% DMSO (dimethyl sulfoxide, Nacalai Tesque, Kyoto, Japan) to 10 mM was added to the well at 5 μM as the final concentration. As the vehicle, DMSO was treated as the final concentration 0.05% (v/v). Marimastat or DMSO was additionally treated every 24 h. After 36 h of a culture in DMEM containing uremic toxins with marimastat or DMSO, cells at 100% confluency were washed with PBS and dissolved in RIPA lysis buffer (Nacalai Tesque, Kyoto, Japan) and protease inhibitor cocktail (04080-11, Nacalai Tesque, Kyoto, Japan). The lysates were centrifuged at 12,000 *rpm* for 15 min, and the supernatant was diluted with 2× SDS sample buffer (Cosmo Bio USA CO., Carlsbad, CA, USA) and denatured at 60° C for 20 min.

### Gelatin zymography

bEnd.3 cells were grown in a 6-well plate. After serum starvation for 12–20 h, the cells were treated with 5% (v/v) mice serum. After 24 h, the cells at 100% confluency were washed with PBS and were used for gelatin zymography. Gelatin zymography was used to measure the levels of matrix metallopeptidase-2 and -9. The supernatants from cell lysates were analyzed using the fluorescein isothiocyanate (FITC)-labeled gelatin zymography kit (Cosmo Bio type) according to the manufacturer’s instructions. Briefly, the supernatant on gel-plates was electrophoresed and the gel was rinsed with washing buffer for 1 h. The diluted mice serum was also loaded as a positive control. The enzyme reaction was performed by incubating the gels with an enzyme reaction buffer at 37° C for 20 h.

### Animals

Seven- to 8-week-old C57BL/6JJcl male mice were purchased from CLEA Japan, Inc., Tokyo, Japan. Animals had access to tap water and their assigned diet under standard lighting conditions (12-h:12-h light–dark cycle). All mice were allowed to acclimate to the conditions of the animal facility for a period of ≥7 days prior to the start of the experiments. Mice were randomly assigned into the experimental groups with the operator blinded to the treatment groups. No mice were excluded.

### Establishment of CKD model rodents displaying a cognitive impairment

Animals were randomly assigned into the control or CKD groups that were fed with a normal chow diet (CL2; CLEA Japan, Inc., Tokyo, Japan) or CL2-containing adenine. CKD in male C57BL/6 mice was induced by a 0.20%-adenine diet for six weeks [19] (Figure 1A). Blood samples were collected from the orbital sinus of mice and serum was extracted after centrifugation (3,000×*g* for 10 min at 4° C). The serum was stored at −80° C. The samples were collected four weeks after reversal to normal diet. Serum urea, creatinine, and renal histology were assessed to verify the establishment of the CKD model. Serum chemistries, including creatinine, and urea nitrogen were measured using a JCA-BM8000 auto analyzer (Nihon Denshi, Tokyo, Japan). We performed a 5/6 nephrectomy as previously described [27]. Eight weeks after the 5/6 nephrectomy or sham-operation, blood or tissue samples were collected.

### Histological tests in kidneys

CKD mouse kidneys were harvested and fixed in 10% Formaldehyde neutral buffer solution (Nacalai Tesque, Kyoto, Japan) for preparation of paraffin embedding. Tissue sections were subjected to Masson’s trichrome staining, and kidney samples were observed and photographed using a BZ-X810 microscope (Keyence, Osaka, Japan).

### Behavioral tests

The behavioral tests used in the present study are all validated and widely used in literature for the evaluation of short-term recognition memory in rodents [20, 21]. In order to prevent the influence of adenine or unnecessary stress, we conducted behavioral tests one week after reversal to normal diet in CKD model mice and serum samples were collected only after the tests. All animals were habituated to the testing room 1 h prior to the test.

### Spontaneous alternation in the Y-maze test

The spontaneous alternation test relies on the tendency of rodents to explore unfamiliar stimuli. Under this assumption, we expect that given the choice to enter only two arms of the maze, animals will enter the less recently visited one. Because functional working memory is required, an alternation is considered a correct choice, whereas a reentry into a previously visited arm is counted as an error. Mice were placed in the Y maze and were allowed to explore for 8 min. A video camera mounted on the wall directly above the box was used to record the testing session for offline analysis. The parameters used to calculate the working memory were the ratio of alterations among the total number of transitions within 8 min. Values that were not over the spontaneous chance level of 50% were interpreted as an indication of impaired working memory.

### Novel object recognition testing

NOR testing is based on the same assumption as the spontaneous alternation test, namely that rodents have an intrinsic tendency to investigate new stimuli [20]. Contrary to the spontaneous alternation test, this paradigm is mainly used as a model for reference memory only and not for spatial working memory [21]. Stimuli consisted of plastic objects that varied in color and shape but were similar in size. A video camera mounted on the wall directly above the box was used to record the testing session for offline analysis. Briefly, all animals were habituated to the empty box measuring 24 cm × 37 cm × 23 cm for 10 min 1 d prior to the test. Twenty-four hours after habituation, mice were placed in the same box in the presence of two identical sample objects and were allowed to explore for 10 min (learning phase). After an intersession interval of 1h, mice were returned to the same box. One of the two objects was replaced with a novel object. Mice were allowed to explore the area for 10 min (test phase). Exploratory behavior was later assessed manually by an experimenter blinded to the treatment group. Exploration of an object was defined as any exploratory behavior triggered by the presence of the object (sniffing, biting, or touching) with the orientation of the nose toward the object within a distance of < 2 cm; however, climbing on objects was excluded. A minimal exploration time for both objects (total exploration time) during the learning and the test phase (~10 s) was used. A discrimination index was calculated as the exploration time of the replaced object minus the exploration time of the unreplaced object divided by the total exploration time of both objects. A higher discrimination index indicated better recognition memory.

We validated whether two types of objects used as “unfamiliar” and “familiar” during the tests were counterbalanced within the experimental groups. We used four wild-type mice in the sex, strain, and age group representative of the experimental mice that were used in the trial above. We allowed them to acclimate in the testing room for 1 h. We then placed each mouse in the box and allowed it to explore the box freely for 10 min. After placing the mice back in their holding cages, we placed 2 different objects near 2 non-release corners such that the objects are counterbalanced in the box and at an area of 5 × 5 cm^2^ from each wall of that corner. After 1 h of retention time, we placed each mouse facing the walls in the release corner and allowed mice to investigate the box and objects freely for 10 min. We analyzed the investigation time of each mouse with each object and confirmed that the mice had no biased interest in or avoidance of one object over the other.

### Sequential protein extraction of the mice brain

Detergent-insoluble tau was extracted according to previously published protocols [22]. Briefly, after mice were anesthetized and sacrificed by cervical dislocation, the hippocampus and cerebral cortex were collected. The tissues were homogenized in 15 volumes of Tris-buffered saline (TBS) buffer containing 50 mM Tris (pH = 7.4), 150 mM NaCl, 1 mM EGTA, 1 mM EDTA, protease inhibitors and phosphatase inhibitors. The homogenates were centrifuged (23,000 r.p.m., 15 min, 4° C) in a TLA100.4 rotor and separated into supernatant (TBS-soluble fractions) and insoluble fractions (pellet). Pellets were resuspended in 15 volumes of 0.32 M sucrose containing 10 mM Tris (pH 7.4), 0.8 M NaCl and 1 mM EGTA and centrifuged (23,000 r.p.m., 15 min, 4° C). Supernatants were collected and treated with 15 volumes of 1% sarkosyl for 1 h at 37° C, and centrifuged at 60,000 r.p.m. for 1 h at 4° C and then separated into supernatant and pellet (sarkosyl-insoluble fraction). Samples from TBS-soluble were diluted with 2× SDS sample buffer (Cosmo Bio USA CO., Carlsbad, CA, USA) and samples from sarkosyl-insoluble fractions were dissolved in 70 μL of SDS sample buffer. Both samples were then boiled for 5 min.

### Measurements of serum levels of IgG

Serum levels of IgG were measured using an automatic biochemical analyzer (JCA-BM8000 series, Japan Electron Optics Laboratory Co., Ltd., Tokyo, Japan) and a turbidimetric immunoassay using reagent “N-Assay TIA IgG-SH Nittobo” (Nittobo Medical Co., Ltd., Tokyo, Japan).

### Proteomics analysis

In the sequential protein extraction process described above, we prepared the samples of each of the soluble and insoluble fractions. Data-independent acquisition (DIA) proteomics analysis was performed by Promega as previously reported [57], using both TBS-soluble fraction and sarkosyl-insoluble fraction derived from the mixed samples of three control mice or CKD mice, respectively. The threshold for protein identification was set such that both protein and peptide false discovery rates were <1%. When the identified peptide count was ≥2, the protein and peptide quantification values were calculated using the Scaffold DIA.

We determined DEGs when fold changes were ≥3.0 in CKD mouse samples as compared to the non-CKD controls. We performed enrichment analyses to determine upregulated and downregulated DEGs with the GO functional analysis, using the Database for Annotation, Visualization, and Integrated Discovery. The data were registered to the public data repository Japan ProteOme STandard Repository/Database (jPOST). The accession numbers are PXD038680 for ProteomeXchange and JPST001941 for jPOST, respectively.

### Administration of Evans blue fluorescence in mice

To evaluate the staining of plasma serum albumin, we utilized the Evans-blue stain because this compound can bind to serum albumin immediately after entering the bloodstream [33]. 2% solution of Evans blue fluorescence in normal saline (4 mL/kg of body weight) was injected intraperitoneally and allowed to circulate for 24 h. Mice were then transcardially perfused with 30 ml of ice-cold PBS followed by 4% paraformaldehyde (PFA), and whole brain preparations were fixed in 4% PFA for 30 min and transferred to a 20% sucrose solution. The samples were washed in PBS, perfused with snap-frozen optimal cutting temperature solution (Sakura Finetek Japan), and stored at −80° C.

### Immunofluorescence study

Cryostat sections (10 μm) of the brain were cut, air dried, and incubated with 1% bovine serum albumin (BSA) in PBS for 30 min to block nonspecific binding of the antibodies. The samples were rinsed in PBS for 5 min and incubated with primary antibody diluted in 0.1% BSA in PBS in a humidified chamber overnight at 4° C. Primary antibodies included goat anti-CD31/PECAM-1 (R&D systems; AF3628; 1:200), rabbit anti-Claudin 5 (Abcam; ab15106; 1:200), rabbit anti-collagen IV (Abcam; ab6586; 1:200), rabbit anti-Tau (phospho S396) (Abcam; ab32057; 1:200), rabbit anti-Beta-Amyloid 1-42 (Sigma-Aldrich; AB5078P; 1:200), and mouse anti-MMP-2 (8B4; Santa Cruz Biotechnology; sc-13595; 1:200). Samples were then washed three times with PBS for 5 min each in the dark and incubated in the following diluted secondary antibodies in 0.1% BSA in PBS for 1 h at room temperature in the dark: donkey anti-goat IgG H&L (Alexa Fluor® 546; 1:200), goat anti-rabbit IgG H&L (Alexa Fluor® 546; 1:200), and goat anti-mouse IgG H&L (Alexa Fluor® 488; 1:200). The IgG immunofluorescence staining was performed with the secondary antibody only. The samples were washed three times with PBS for 5 min each in the dark, mounted coverslip with a drop of mounting medium, and stored in the dark at 4° C. Images of the stained sections were captured using a Nikon AX confocal microscopy with Nikon Spatial Array Confocal (NSPARC, Nikon, Tokyo, Japan) detector (Figures 3, 4, and Supplementary Figure 2) or BZ-X810 microscope (Supplementary Figure 3). CD31 positive regions were measured using ImageJ software (National Institutes of Health).

### Immunoblotting

The concentration of protein extracts taken from the mouse brain samples was determined via the Bradford ULTRA kit (Cosmo Bio, Co, LTD, Tokyo, Japan), using BSA standards. Mouse serum was mixed with 2× SDS sample buffer and boiled for 5 min. Protein extracts were separated by SDS–PAGE, electrically transferred to a nitrocellulose membrane, and probed with the following primary antibodies: rabbit anti-Tau (phospho S396) (Abcam; ab32057; 1:1,000), rabbit anti-Tau (phospho S396) (Abcam; ab109390; 1:1,000), mouse anti-Tau (Tau46) (Cell Signaling Technology; #4019; 1:1,000), rabbit anti-CD31 (Abcam; ab28364; 1:1,000), rabbit anti-Claudin 5 (Abcam; ab15106; 1:1,000), rabbit anti-MMP2 (Abcam; ab92536; 1:1,000), mouse anti-MMP2 (8B4; Santa Cruz Biotechnology; sc-13595; 1:1,000), rabbit anti-fibronectin (Abcam; ab2413; 1:1,000), rabbit anti-alpha smooth muscle actin (Abcam; ab5694; 1:1,000), and mouse anti-GAPDH (Santa Cruz Biotechnology; sc-32233; 1:1,000). Alkaline phosphatase-conjugated anti-IgG antibodies (Promega, Madison, WI, USA) were used as secondary antibodies. The band densities of the proteins were quantified using ImageJ software (National Institutes of Health). The IgG Western blot was performed with the secondary antibody only.

### Quantitative real-time PCR

Total RNA was isolated using Sepasol-RNAI Super G (Nacalai Tesque, Kyoto, Japan) according to the manufacturer’s instructions. Total RNA extracted from the mouse hippocampus was reverse transcribed using ReverTra Ace (TOYOBO; Tokyo, Japan). Quantitative real-time PCR was performed on a Thermal Cycler Dice Real Time System Lite TP700 (Takara Bio Inc., Otsu, Japan) using TB Green Premix Ex Taq II (Takara Bio Inc.). The primers used in this study are described in Supplementary Table 1. PCR amplification consisted of 45 cycles at 95° C for 5 s and 55° C for 10 s and 72° C for 20 s after an initial denaturation step at 95° C for 3 s. The 2^−ΔΔct^ method was used to compare mRNA expression levels, and the relative expression levels of each target gene were normalized with GAPDH as an internal control.

### Analysis of CKD patient samples

We enrolled 980 adult patients with dialysis-independent CKD from two single-center, prospective observational cohorts (Supplementary Table 2). In the first cohort, inclusion of participants was based on the following criteria: (1) inpatients or outpatients at our university hospital between October 2020 and December 2021, (2) age from 18 to 74 years, and (3) dialysis-independent CKD according to the KDIGO classification [42]. GFR was estimated using the three-variable Japanese GFR equation developed by the Japanese Society of Nephrology [43]: eGFR (mL/min per 1.73 m^2^) = 194 × serum creatinine^−1.094^ × age^−0.287^ (if female, × 0.739).

We also recruited patients from a previously established cohort (CKD-ROUTE study) [41]. This cohort originally enrolled patients with CKD stages 2–5 at the Tokyo Medical and Dental University Hospital and its 15 affiliated, larger than midsized clinical centers located in the greater Tokyo metropolitan area. Participants were eligible for inclusion if they: (1) newly visited or were newly referred to the participant nephrology centers from October 2010 to December 2011; (2) were over 20 years of age; and (3) had CKD stages 2–5 according to the KDIGO classification [42].

From these two cohorts, patients were excluded if they lacked information on BMI, WBC, hemoglobin, platelet count, serum albumin, serum urea nitrogen, creatinine and eGFR, serum sodium, potassium, chloride, calcium, and phosphate. Definition of cognitive impairment was based on a diagnosis of dementia, a score of ≤20 on the revised Hasegawa’s Dementia Scale, or a Mini-Cog score of ≤4 on medical records.

### Statistical analysis

Statistical significance between the two groups was evaluated using an unpaired t test or Wilcoxon signed-rank test. Normality was assessed with the Shapiro–Wilk test. When variables were nonparametric, we used the Wilcoxon signed-rank test. Statistical analyses among more than two groups were performed using one-way ANOVA followed by Turkey’s post-hoc test for parametric variables or Kruskal–Wallis test followed by Dunn’s multiple comparisons test for nonparametric variables. The Jonckheere–Terpstra trend test was used to indicate potential protein expression trends in response to uremic solutes’ concentration gradient. To investigate the variables associated with a risk for cognitive impairment, we performed cross-sectional analyses using baseline characteristics and data of the two CKD cohorts with multivariable logistic regression models adjusting for confounding factors. Data are presented as mean ± standard deviation of the mean (S.D.). Statistical analyses were performed using EZR (Saitama Medical Center, Jichi Medical University, Saitama, Japan) and GraphPad Prism 9 (GraphPad Software, Inc., San Diego, CA, USA). Only within-test corrections were made. The analyses were also performed in a randomized and blinded fashion, where appropriate. A value of *P* < 0.05 was considered statistically significant.

### Data availability

The proteomic data were registered to the public data repository Japan ProteOme STandard Repository/Database (jPOST). The accession numbers are PXD038680 for ProteomeXchange and JPST001941 for jPOST, respectively. The other data underlying this article will be shared on reasonable request to the corresponding author.

## Supplementary Material

Supplementary Figures

Supplementary Tables
