# Supplementary material for: Chronic kidney disease causes blood-brain barrier breakdown via urea-activated matrix metalloproteinase-2 and insolubility of tau protein
Source: Aging (Albany NY). 2023 Oct 25;15(20):10972–95. doi: 10.18632/aging.205164 (PMC10637825; doi:10.18632/aging.205164)
Supplement: Supplementary Figures [file aging-15-205164-s001.pdf]

SUPPLEMENTARY FIGURES

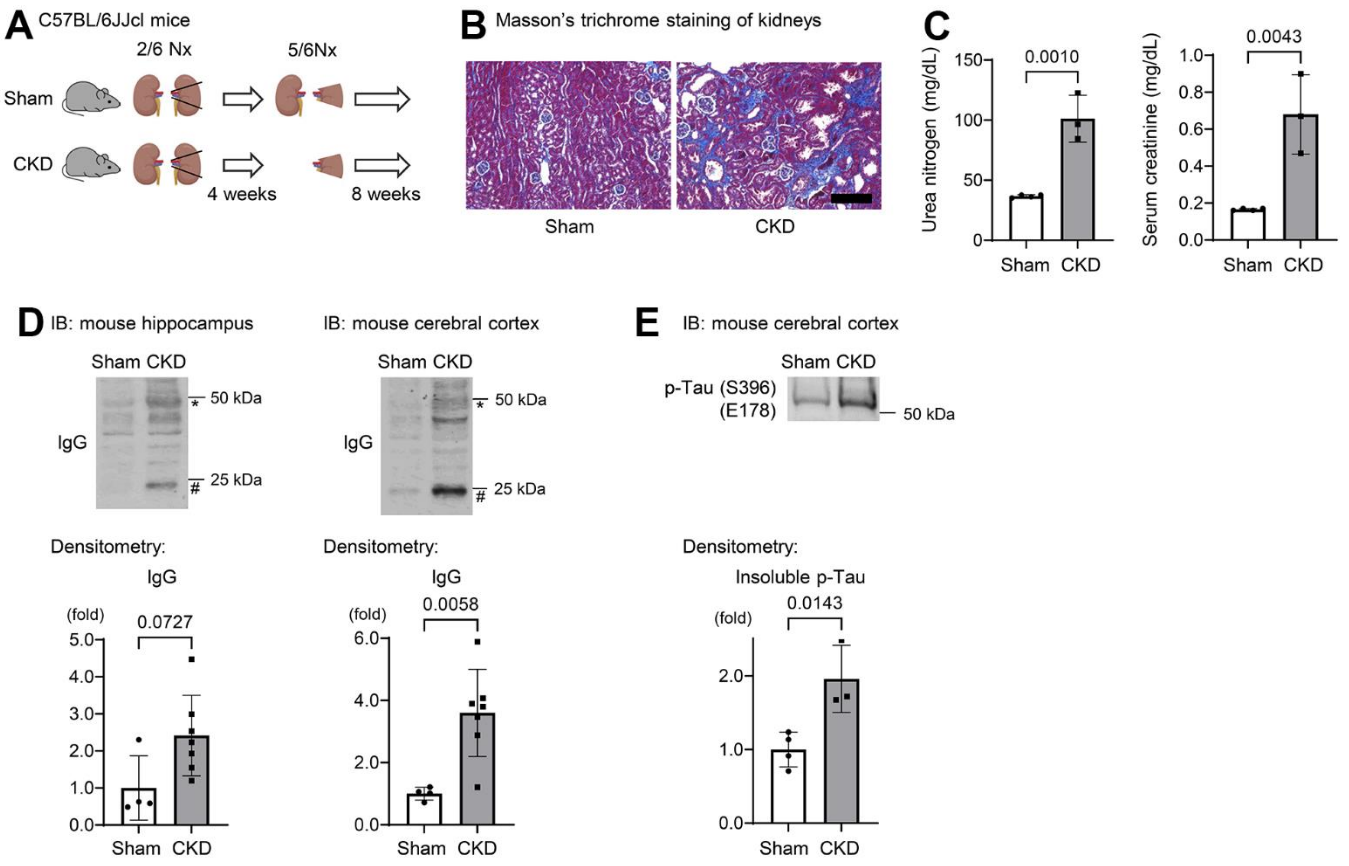

Supplementary Figure 1. A model of CKD established by a 5/6 nephrectomy causes a BBB breakdown and the accumulation of insoluble tau. (**A**) Establishment of a CKD model in mice by 5/6 nephrectomy in C57BL/6JJcl mice. (**B**) Masson's Trichrome staining showed tubulointerstitial fibrosis in the kidneys after 5/6 nephrectomy. Scale bar = 100 μm. (**C**) Serum urea nitrogen and creatinine, indicating uremic solutes, were elevated in CKD (*n* = 4 in the sham-operation group; *n* = 3 in the CKD group). (**D**) Western blotting showed CKD increased expressions of IgG in hippocampus and neocortex tissues of CKD mice (*n* = 4 in the sham-operation group; *n* = 7 in the CKD group). (**E**) Phosphorylated tau in the sarkosyl-insoluble (aggregated) fraction in CKD mice was elevated compared with the control group (*n* = 4 in the sham-operation group; *n* = 3 in the CKD group). Data are presented as mean ± standard deviation of the mean. Normality was assessed with the Shapiro–Wilk test. Statistical significance between the two groups was evaluated using an unpaired t test or Wilcoxon signed-rank test. When variables were nonparametric, we used the Wilcoxon signed-rank test. *P* < 0.05 was considered statistically significant. CKD, chronic kidney disease.

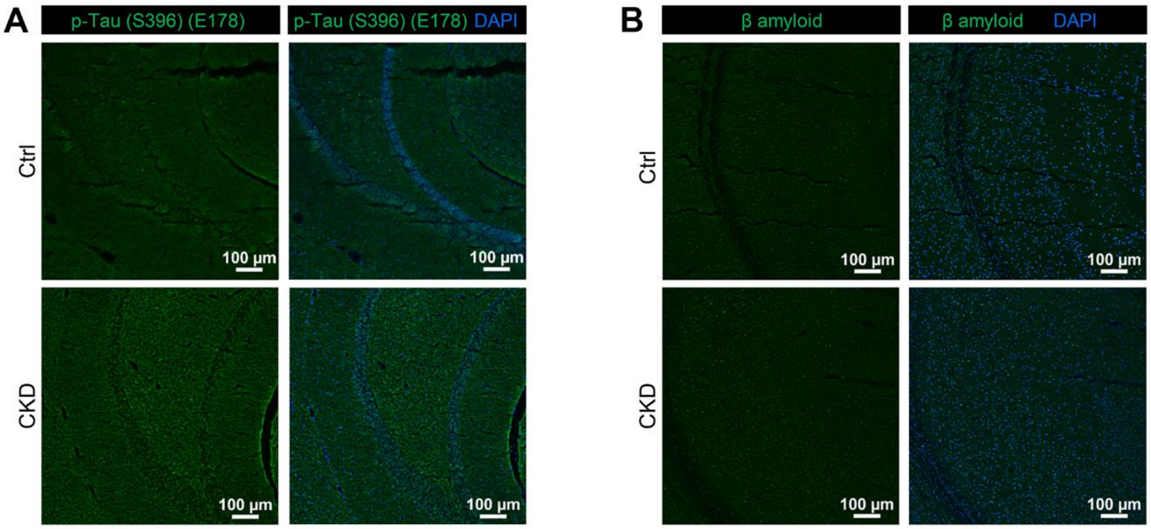

Supplementary Figure 2. Phosphorylated tau and β amyloid in the hippocampus tissue of CKD mice and control mice. (**A**) Immunofluorescence staining revealed no obvious phosphorylated tau deposits in the hippocampus tissue of CKD model mice and control mice. (**B**) Immunofluorescence staining revealed no depositions of β amyloid in the hippocampus of CKD mice or control mice. CKD, chronic kidney disease.

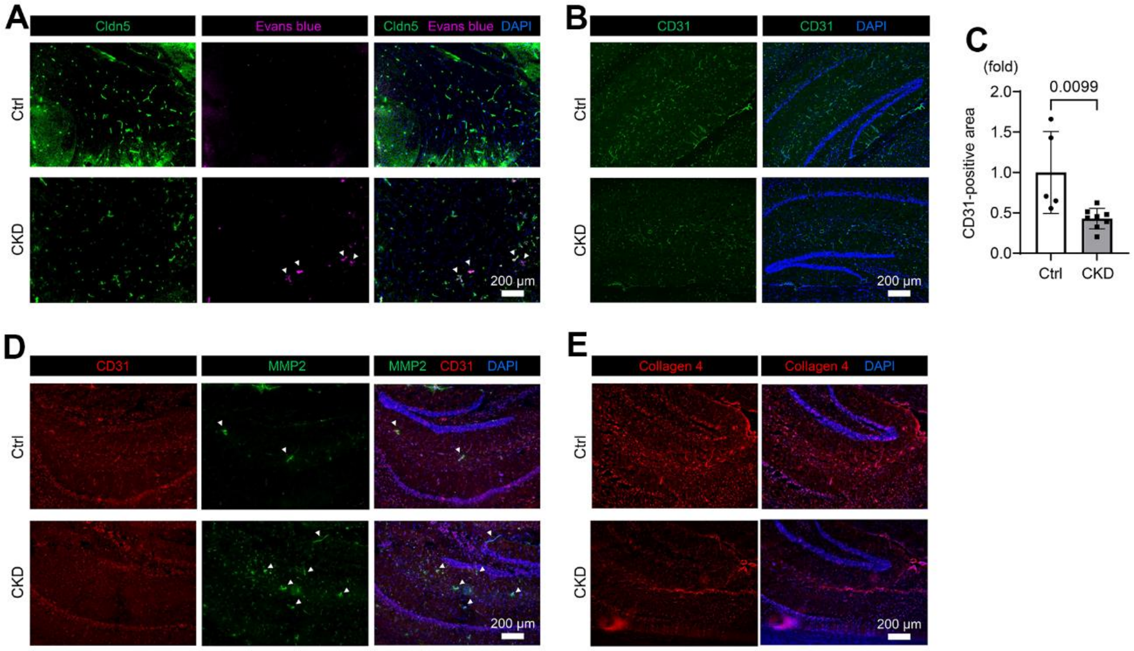

Supplementary Figure 3. The downregulations of tight junction, adherens-junction, and basement membrane proteins in the CKD mouse brain. (**A**) Immunofluorescence indicating the staining pattern of claudin-5 in the hippocampus tissue of CKD mice compared to the control group. Leakage of Evans blue fluorescence was only seen in CKD mice (white arrowhead). (**B**) Immunofluorescence study showing that the adherens-junction protein platelet/endothelial cell adhesion molecule-1 (PECAM-1)/CD31 expression was decreased in the hippocampus of CKD mice compared to the control group. (**C**) CD-31-positive area was quantified and was found decreased in CKD (n = 5 in the control group; n = 8 in the CKD group). (**D**) Immunofluorescence study showing that the protein expression of MMP2 (white arrowhead) was increased in the hippocampus tissue of CKD mice, in contrast to the decreased CD31 expression. (**E**) Immunofluorescence study showing that the expression of collagen IV, a substrate of MMP2, a known collagenase that is a key protein of the basement membrane, was suppressed in the CKD hippocampus tissues. Data are presented as mean ± standard deviation of the mean. Normality was assessed with the Shapiro–Wilk test. Statistical significance between the two groups was evaluated using an unpaired t test. $P < 0.05$ was considered statistically significant. CKD, chronic kidney disease; MMP2, matrix metalloproteinase-2.

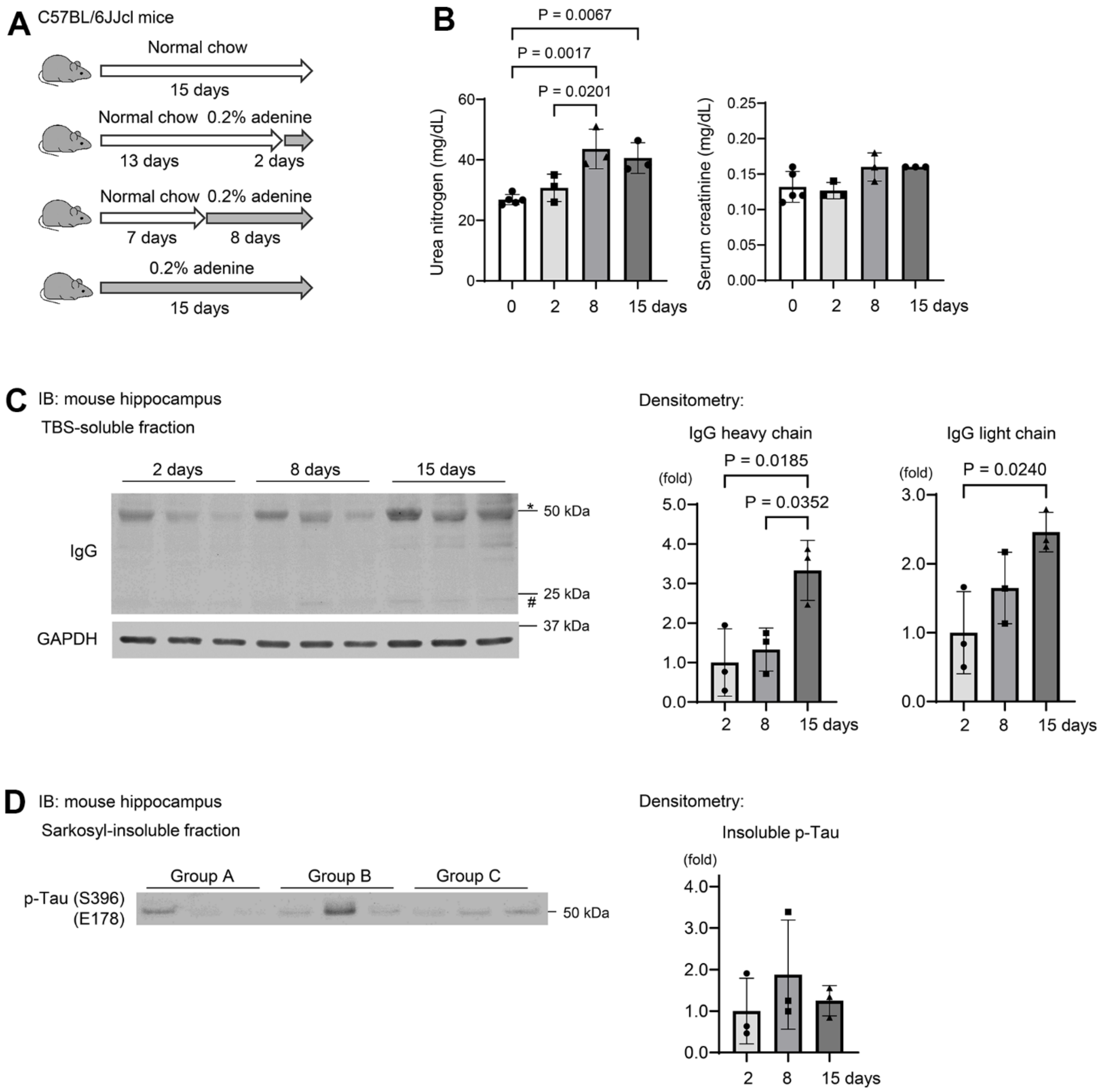

Supplementary Figure 4. A leakage of IgG to the mouse hippocampus following elevated serum urea preceded the accumulation of insoluble tau in kidney impairment. (**A**) Short-term kidney impairment models in mice by providing wild-type C57BL/6JJcl mice a diet containing 0.20% adenine with different allocation periods (2 days, 8 days, and 15 days). (**B**) Serum urea nitrogen and serum creatinine levels in short-term kidney impairment models (*n* = 3 per group). (**C**) Western blotting showed the increased soluble IgG heavy and light chains of mouse hippocampus 15 days after initiating 0.20%-adenine diet (*n* = 3 per group). * represents IgG heavy chain, and # represents the IgG light chain. (**D**) Insoluble phosphorylated tau levels were not increased during the time course (*n* = 3 per group). Data are presented as mean ± standard deviation of the mean. Statistical analyses among multiple groups were performed using one-way ANOVA, followed by Turkey's post-hoc test. $P < 0.05$ was considered statistically significant.
