# Supplementary material for: Chronic kidney disease causes blood-brain barrier breakdown via urea-activated matrix metalloproteinase-2 and insolubility of tau protein
Source: Aging (Albany NY). 2023 Oct 25;15(20):10972–95. doi: 10.18632/aging.205164 (PMC10637825; doi:10.18632/aging.205164)
Supplement: Supplementary Tables [file aging-15-205164-s002.pdf]

SUPPLEMENTARY TABLES

Supplementary Table 1. Primer sequences used for the experiments.

Gene	Species	Forward primer (5'→3')	Reverse primer (5'→3')
Gapdh	Mus	CGTGGAGTCTACTGGTGTCTTCAC	CGGAGATGATGACCCTTTTGGC
Ighg1	Mus	GACGGGGAGCTGGACGGGCTCTG	CACCACCGAGGAGAAGATCCAC

Mus, *Mus musculus*.

Supplementary Table 2. Characteristics of the study participants.

Characteristic	Whole group
N	980
Age, year	70 (61–77)
Female	293 (30)
Body mass index, kg/m^2	23.2 (21.0–26.1)
Diabetes mellitus	369 (38)
Cardiovascular disease	274 (28)
White blood cell count, /μL	6,200 (5,153–7,498)
Hemoglobin, g/dL	11.8 (10.2–13.4)
Platelet count, $\times 10^4$/μL	20.6 (16.6–25.0
Serum sodium, mEq/L	140 (138–141)
Serum potassium, mEq/L	4.5 (4.2–4.9)
Serum chloride, mEq/L	106 (104–108)
Serum calcium[a], mg/dL	9.1 (8.7–9.5)
Serum phosphate, mg/dL	3.5 (3.1–4.0)
Serum albumin, g/dL	4.0 (3.5–4.3)
Serum creatinine, mg/dL	1.8 (1.2–2.8)
Estimated GFR, mL/min/1.73 m^2	29 (17–45)
Urea nitrogen, mg/dL	28 (19–40)

Data are numbers (percentiles) or medians (interquartile range). GFR, glomerular filtration rate; IQR, interquartile range.
[a]Serum calcium level was corrected when under 4.0 using the formula: serum corrected calcium level = total calcium level + (4.0–serum albumin).
